# Epitaxial growth and superconducting properties of thin-film PdFe/VN and VN/PdFe bilayers on MgO(001) substrates

**DOI:** 10.3762/bjnano.11.65

**Published:** 2020-05-15

**Authors:** Wael M Mohammed, Igor V Yanilkin, Amir I Gumarov, Airat G Kiiamov, Roman V Yusupov, Lenar R Tagirov

**Affiliations:** 1 Kazan Federal University, Kremlyovskaya str. 18, 420008 Kazan, Russia; 2 E. K. Zavoisky Physical-Technical Institute, FRC Kazan Scientific Centre of RAS, 420029 Kazan, Russia

**Keywords:** epitaxial growth, epitaxial superconductor–ferromagnet heterostructure, palladium–iron alloy (PdFe), vanadium nitride (VN), superconducting spintronics

## Abstract

Single-layer vanadium nitride (VN) and bilayer Pd_0.96_Fe_0.04_/VN and VN/Pd_0.92_Fe_0.08_ thin-film heterostructures for possible spintronics applications were synthesized on (001)-oriented single-crystalline magnesium oxide (MgO) substrates utilizing a four-chamber ultrahigh vacuum deposition and analysis system. The VN layers were reactively magnetron sputtered from a metallic vanadium target in Ar/N_2_ plasma, while the Pd_1−_*_x_*Fe*_x_* layers were deposited by co-evaporation of metallic Pd and Fe pellets from calibrated effusion cells in a molecular beam epitaxy chamber. The VN stoichiometry and Pd_1−_*_x_*Fe*_x_* composition were controlled by X-ray photoelectron spectroscopy. In situ low-energy electron diffraction and ex situ X-ray diffraction show that the 30 nm thick single-layer VN as well as the double-layer VN(30 nm)/Pd_0.92_Fe_0.08_(12 nm) and Pd_0.96_Fe_0.04_(20 nm)/VN(30 nm) structures have grown cube-on-cube epitaxially. Electric resistance measurements demonstrate a metallic-type temperature dependence for the VN film with a small residual resistivity of 9 μΩ·cm at 10 K, indicating high purity and structural quality of the film. The transition to the superconducting state was observed at 7.7 K for the VN film, at 7.2 K for the Pd_0.96_Fe_0.04_/VN structure and at 6.1 K for the VN/Pd_0.92_Fe_0.08_ structure with the critical temperature decreasing due to the proximity effect. Contrary to expectations, all transitions were very sharp with the width ranging from 25 mK for the VN film to 50 mK for the VN/Pd_0.92_Fe_0.08_ structure. We propose epitaxial single-crystalline thin films of VN and heteroepitaxial Pd_1−_*_x_*Fe*_x_*/VN and VN/Pd_1−_*_x_*Fe*_x_* (*x* ≤ 0.08) structures grown on MgO(001) as the materials of a choice for the improvement of superconducting magnetic random access memory characteristics.

## Introduction

Since its invention, rapid single-flux quantum (RSFQ) logic [1–2] based on superconducting digital electronics has been seriously considered as an alternative to semiconductor electronics for supercomputing applications [3–5]. Merging it with magnetism [6–8] has given a birth to superconducting spintronics [9–10]. The latter concept was implemented in the US Cryogenic Computing Complexity (C3) Program [11–13] with the goal “to demonstrate a small-scale computer based on superconducting logic and cryogenic memory that is energy-efficient, scalable and able to solve interesting problems”, opening prospects of reaching 100 PFLOPS/s with about 200 kW of electric power consumption including the cryogenic cooling. Niobium-based Josephson junction technology is currently implied to be used for the logics fabrication, however, hybrid Josephson junctions incorporating magnetic components are also considered for the mainframe computation components [9,14–19], and cache and main memories [8,20–25]. It is argued that the use of magnetic Josephson junctions in single-flux quantum electronics significantly reduces the number of junctions and interconnects in the circuits [26] and also has other important advantages such as wide operation margins and low bit-error rate [27]. The magnetic material has to be magnetically soft, tunable and weak in the sense of small spin-polarization of the conduction band [10,28]. The latter provides a large superconducting coherence length and hence bypasses a necessity to deposit flat, nanometer-thick continuous layers expected for strong elemental ferromagnets. A combination of niobium as a superconductor with a Pd_1−_*_x_*Fe*_x_* alloy as a soft and weak ferromagnet was considered as material of choice for superconducting magnetic random access memories (MRAM) [8,29–30]. However, no further developments towards a prototype using a Pd_1−_*_x_*Fe*_x_* alloy have been demonstrated. There are indications of non-homogeneous, nanoclustered magnetism in Pd_0.99_Fe_0.01_ films grown on niobium [31], which may cause a shortening of the spin-memory length [32] and a reduction of the Josephson critical current.

In general, the metallic Nb lattice (body-centered cubic with *a*_Nb_ = 329.4 pm) poorly matches that of the palladium-rich Pd_1−_*_x_*Fe*_x_* alloys (face-centered cubic with *a*_0_ = 389 pm). Therefore, a good crystallinity of the layer stack can hardly be expected. In the resulting polycrystalline films, crystallite boundaries and crystal lattice imperfections can lead to the segregation of iron impurities and to nanoclustering of the alloy. Following the development of a way to grow single-crystalline, magnetically homogeneous epitaxial Pd_1−_*_x_*Fe*_x_* films on MgO(001) single-crystalline substrates [33], we propose fully epitaxial Pd_1−_*_x_*Fe*_x_*/VN and VN/Pd_1−_*_x_*Fe*_x_* (*x* ≤ 0.08) building blocks as an alternative choice for superconducting MRAM materials, in which vanadium nitride (VN) serves as the superconductor. The magnetic anisotropies of a 20 nm thick Pd_0.96_Fe_0.04_ film of the first-generation epitaxial sample of VN/Pd_0.96_Fe_0.04_ on MgO(001) were studied by using a ferromagnetic resonance technique in [34].

## Results and Discussion

### Sample preparation

Single-crystalline MgO(001) (henceforth designated MgO) epi-polished substrates (CRYSTAL GmbH, Germany) with a size of 10 × 5 × 0.5 mm^3^ were annealed at 800 °C for 5 min in the ultrahigh vacuum (UHV) molecular beam epitaxy (MBE) chamber with a residual pressure below 10^−10^ mbar (SPECS, Germany). Then, depending on the desired structure, either the Pd_1−_*_x_*Fe*_x_* alloy layer or the VN layer was deposited. The Pd_1−_*_x_*Fe*_x_* layers were grown by means of UHV MBE following a three-step procedure described in detail in [33]. Metallic Pd (99.95% purity, EVOCHEM GmbH, Germany) and Fe (99.97% purity, ChemPur GmbH, Germany) were co-evaporated from the pre-calibrated high-temperature effusion cells to obtain the desired Pd_1−_*_x_*Fe*_x_* composition.

Vanadium nitride layers were synthesized by using reactive DC magnetron sputtering (MS) in the UHV chamber with a base pressure of *p* ≤ 5 × 10^−10^ mbar (BESTEC, Germany). During this process, the substrate had a temperature of 500 °C. A mixture of high-purity (99.9999%) argon (Ar) from a gas chromatography purification system and high-purity (99.9999%) nitrogen (N_2_) at a composition of Ar/N_2_ = 60:40 was used as plasma gas for the reactive synthesis of VN. During the deposition process, the pressure of the Ar/N_2_ gas mixture in the chamber was automatically kept at 6 × 10^−3^ mbar. A metallic vanadium disk of 99.95% purity (GIRMET Ltd, Russia) was used as a target. The magnetron power was 50 W, the distance between the target and the substrate was 20 cm, and the deposition rate was 0.2 nm/min.

To grow heterostructures, the samples on the molybdenum holder were moved without breaking vacuum via the UHV transfer line between the MBE and MS deposition chambers as well as the analysis chamber (SPECS, Germany).

To perform a comparative study allowing to see only the proximity effect of the ferromagnetic layer on the properties of the superconducting VN layer, the latter was deposited in one run for all studied samples. To do this, we mounted two 10 × 5 × 0.5 mm^3^ MgO substrates close and parallel to each other on the sample holder and used a system of two orthogonal shutters in the MBE chamber. After depositing a 20 nm thick Pd_0.96_Fe_0.04_ layer onto one substrate (with the second being blocked by the shutter), the sample holder was moved to the magnetron chamber, and a 30 nm layer of VN was grown on both substrates. Then, the holder was moved back to the MBE chamber, and a 12 nm thick Pd_0.92_Fe_0.08_ layer was deposited to a half of both samples using the second shutter. The thicknesses of the Pd_1−_*_x_*Fe*_x_* layers were adjusted to possess identical magnetic moments. In situ tests of crystallinity, VN stoichiometry and resulting composition of Pd_1−_*_x_*Fe*_x_* were taken at each deposition step using low-energy electron diffraction (LEED) and X-ray photoelectron spectroscopy (XPS). Finally, all structures were capped with 10 nm layer of undoped Si by magnetron sputtering to prevent sample deterioration. Thus, a VN film and stacks of Pd_0.96_Fe_0.04_/VN and VN/Pd_0.92_Fe_0.08_ (the first component in a stack being directly deposited to MgO) have been obtained with the identical properties of the VN layer in each sample.

### Crystallinity and epitaxial growth

**Figure 1:**
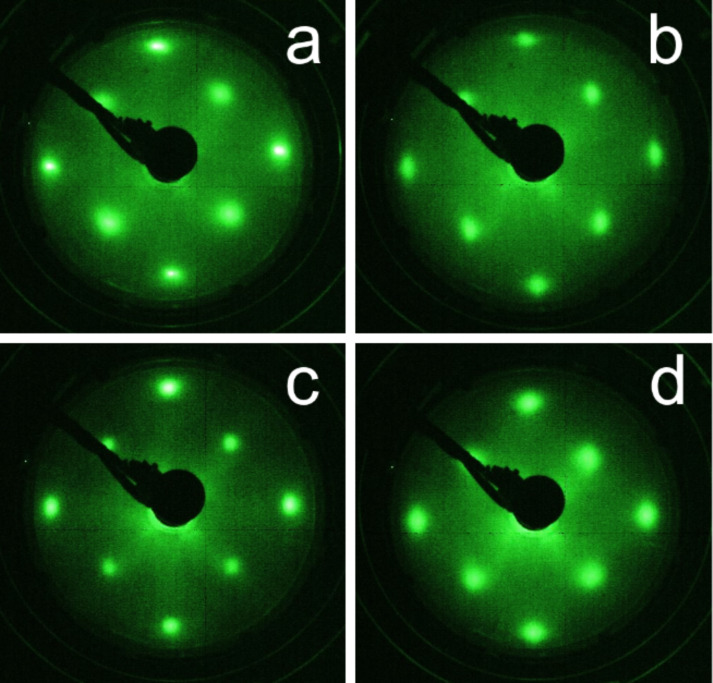
LEED patterns of (a) pristine MgO annealed at 800 °C, (b) the VN film, (c) the VN/Pd_0.92_Fe_0.08_ and (d) Pd_0.96_Fe_0.04_/VN structures on the MgO(001) substrate. All patterns were taken at an electron energy of 140 eV.

The crystallinity and the epitaxial growth of the thin films were examined in situ by using LEED (SPECS, Germany). LEED images were taken of the pristine MgO(001) substrate after annealing (Figure 1a), after the deposition of VN(30 nm) on MgO (Figure 1b), after the deposition of Pd_0.92_Fe_0.08_ on VN (Figure 1c) and after the deposition of VN on Pd_0.96_Fe_0.04_ (Figure 1d). Figure 1b indicates that the individual VN thin film has grown cube-on-cube epitaxially (for an individual Pd_1−_*_x_*Fe*_x_* film see the full crystallinity analysis in [33]). Figure 1c,d shows that the Pd_0.96_Fe_0.04_/VN and VN/Pd_0.92_Fe_0.08_ heterostructures are pass-through epitaxial. This is, first of all, due to the good lattice match between MgO, VN and Pd: *a*_MgO_ = 421.2 pm, *a*_VN_ = 413.7 pm [35] and *a*_Pd_ = 389.1 pm. Thus, the lattice mismatch between MgO and VN is only about 1.7%, and between Pd and VN it is as small as 5.95%.

**Figure 2:**
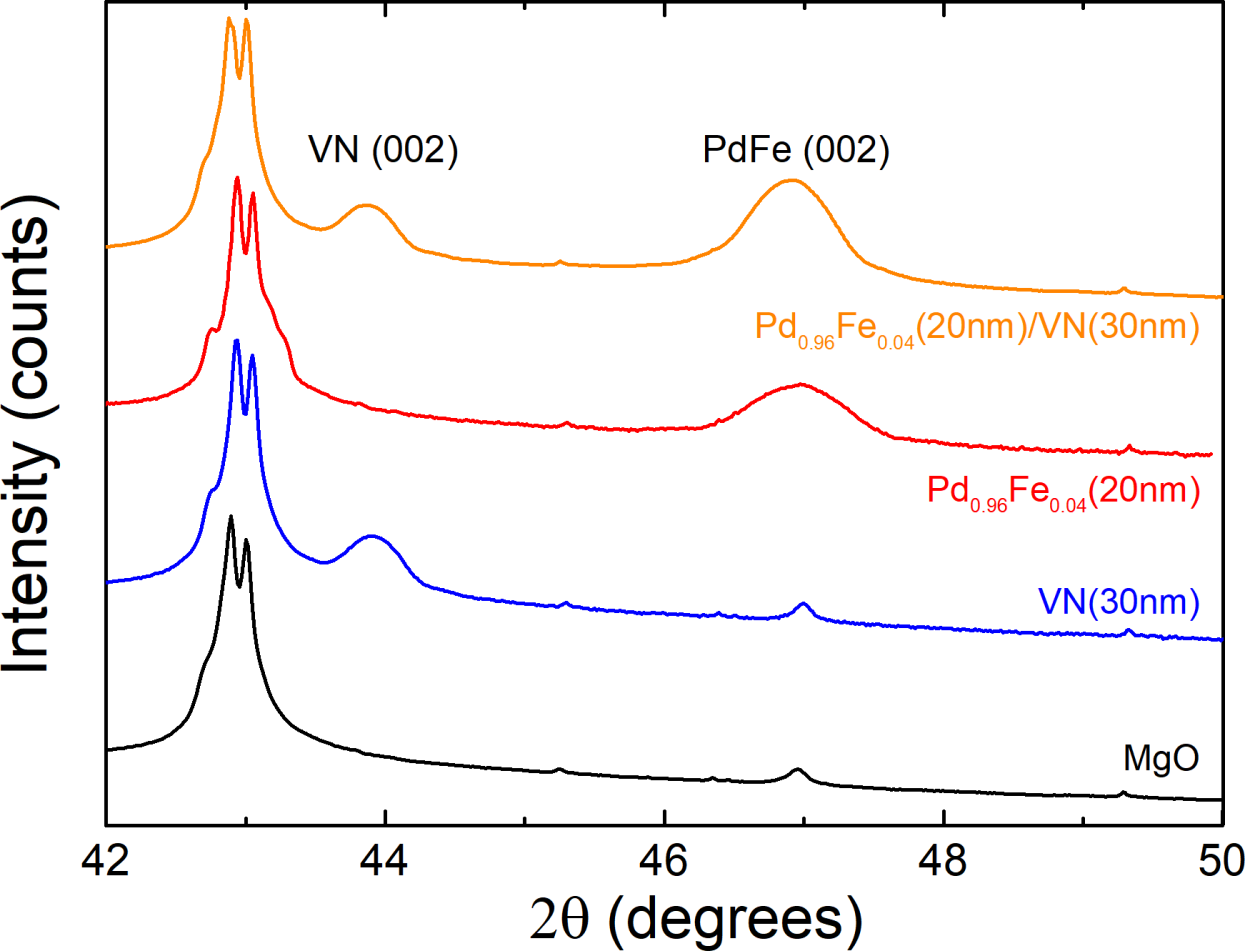
XRD patterns of pristine MgO substrate, VN, Pd_0.96_Fe_0.04_ (prepared in a separate deposition experiment) and Pd_0.96_Fe_0.04_/VN.

The in situ LEED analysis was corroborated with ex situ X-ray diffraction (XRD, BRUKER D8, Germany) measurements using Cu Kα (λ = 1.5418 Å) radiation in the Bragg–Brentano geometry with a scanning rate of 0.002°/s in the 2θ range from 17° to 82° and a step width of 0.0153°. Room-temperature XRD patterns of the pristine MgO(001) substrate, the VN thin film on MgO, Pd_0.96_Fe_0.04_ on MgO and the Pd_0.96_Fe_0.04_/VN heterostructure are shown in Figure 2. The θ–2θ scans clearly indicate the single-crystalline structure of the VN and Pd_0.96_Fe_0.04_ thin films and of the Pd_0.96_Fe_0.04_/VN heterostructures. The (002) reflex of the MgO substrate, the (002) reflex of the VN film (30 nm), and the (002) reflex of the Pd_0.96_Fe_0.04_ (20 nm) film can be easily identified.

The significant peak broadening of the diffraction maxima of VN and Pd_0.96_Fe_0.04_ is primarily due to small coherent scattering range τ along the normal to the film plane (Scherrer broadening); XRD data with accounting for the instrument function [33] yields estimates of τ ≈ 22.0 nm for Pd_0.96_Fe_0.04_, τ ≈ 12.6 nm for Pd_0.92_Fe_0.08_ and τ ≈ 30.4 nm for VN, which agree quantitatively with the film thickness values *d*(Pd_0.96_Fe_0.04_) ≈ 21.5 nm, *d*(Pd_0.92_Fe_0.08_) ≈ 12.5 nm and *d*(VN) ≈ 29.8 nm, respectively, measured ex situ with a BRUKER DektakXT stylus profiler by using the shadow mask method. Thus, LEED and XRD measurements confirm that the VN thin film and the Pd_0.96_Fe_0.04_/VN and VN/Pd_0.92_Fe_0.08_ heterostructures have grown cube-on-cube epitaxially and that all samples are single crystalline.

### Stoichiometry and chemical composition

**Figure 3:**
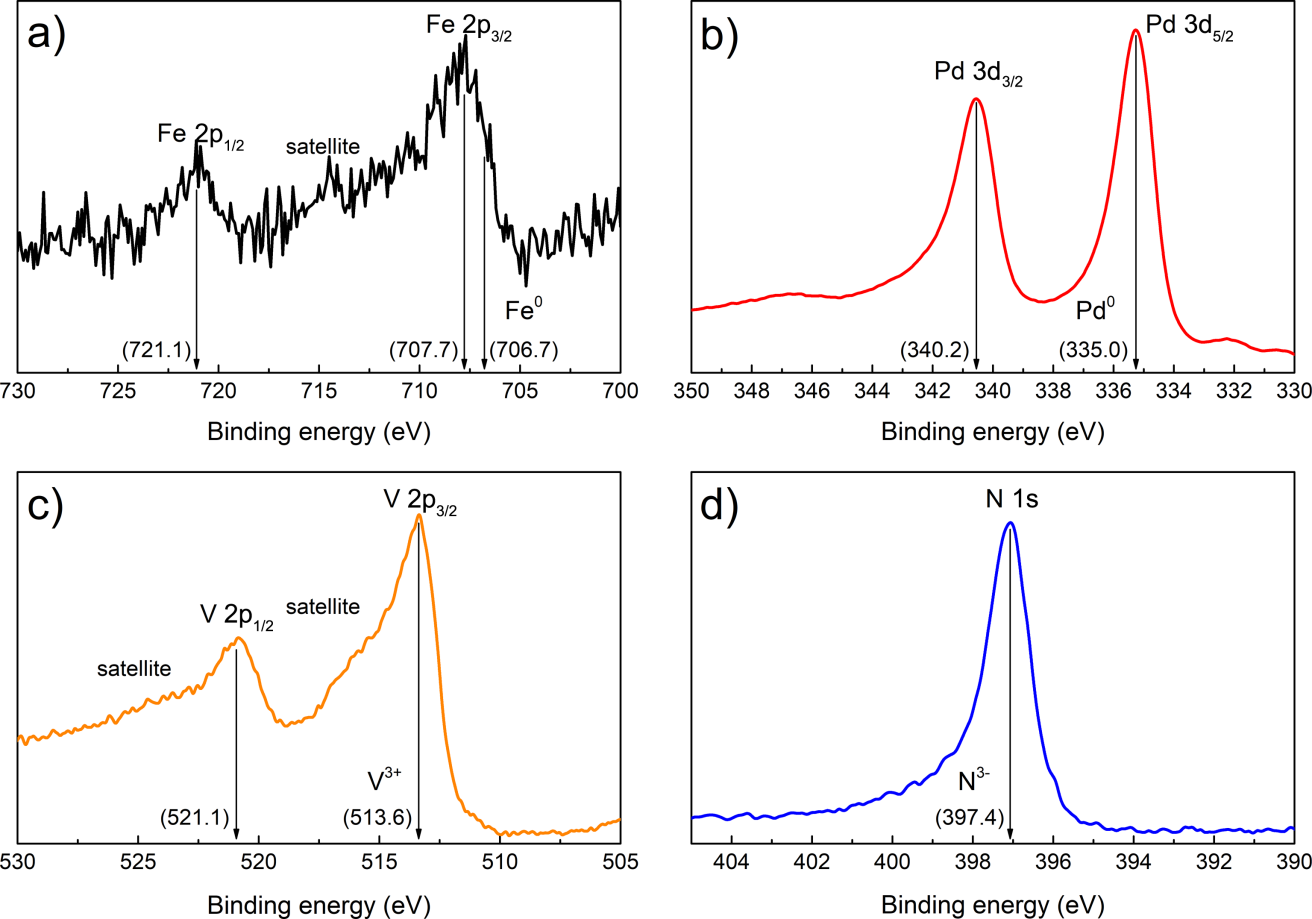
In situ XPS spectra of (a) Fe and (b) Pd of the VN/Pd_0.92_Fe_0.08_ sample, and of (c) V and (d) N of the VN film.

The stoichiometry and chemical composition of the VN and the Pd_1−_*_x_*Fe*_x_* layers were analyzed in situ using XPS. The measurements were carried out in the UHV analysis chamber (base pressure *p* < 3 × 10^−10^ mbar) equipped with a Mg Kα X-ray source operated at 12.5 kV and 250 W, and a Phoibos-150 hemispherical energy analyzer (all from SPECS, Germany). Figure 3a,b shows the XPS spectra of the as-deposited VN/Pd_0.92_Fe_0.08_ thin film heterostructure. The binding energies of the Fe 2p_1/2_, Fe 2p_3/2_, and Pd 3d_3/2_ and Pd 3d_5/2_ states are 721.0, 707.7, and 340.2 and 335.0 eV, respectively, which agrees well with literature data [33,36].

Figure 3c,d shows the XPS spectra of the VN thin film on MgO. The binding energies of the V 2p_1/2_, V 2p_3/2_ and N 1s states are 521.1, 513.6 and 397.4 eV, respectively, which are very close to that given in the literature for crystalline VN [37–38]. The presence of a characteristic satellite at a binding energy of ca. 515 eV is a fingerprint of V in a nitride compound [37]. The chemical composition of the as-grown VN and Pd_1−_*_x_*Fe*_x_* layers was analyzed with the CasaXPS software [39]. According to the XPS data, the stoichiometry of synthesized layers was Pd/Fe = 96:4, V/N = 52.5:47.5 and Pd/Fe = 92:8, respectively, with an accuracy of ±0.5%. Neither impurities nor surface contaminations were detected (compare with [40]). All recorded high-resolution XPS spectra of VN and Pd_1−_*_x_*Fe*_x_* films were calibrated to the binding energies of crystalline VN at 513.6 eV and of metallic Pd at 335.0 eV [33,37], respectively.

**Figure 4:**
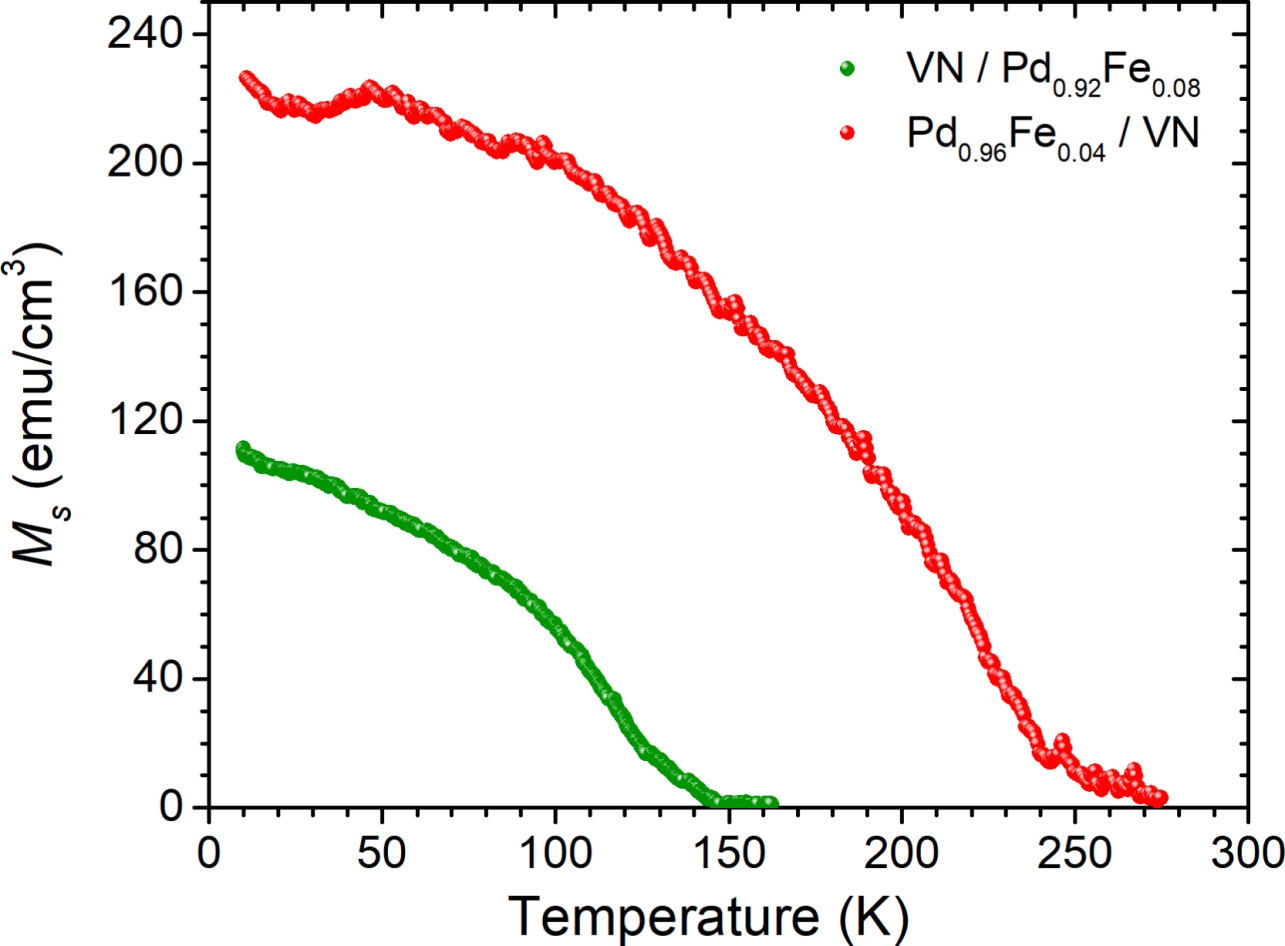
Saturation magnetization *M*_s_(*T*) as a function of the temperature of the Pd_0.96_Fe_0.04_/VN (green symbols) and VN/Pd_0.92_Fe_0.08_ (red symbols) heterostructures measured in a magnetic field of 200 Oe.

Magnetic moment measurements shown in Figure 4 confirm the composition of Pd_0.96_Fe_0.04_ and Pd_0.92_Fe_0.08_ through the ferromagnetic transition temperature *T*_C_ ≈ 125 K and *T*_C_ ≈ 240 K, respectively [41].

### Temperature dependence of resistance and superconducting transition

**Figure 5:**
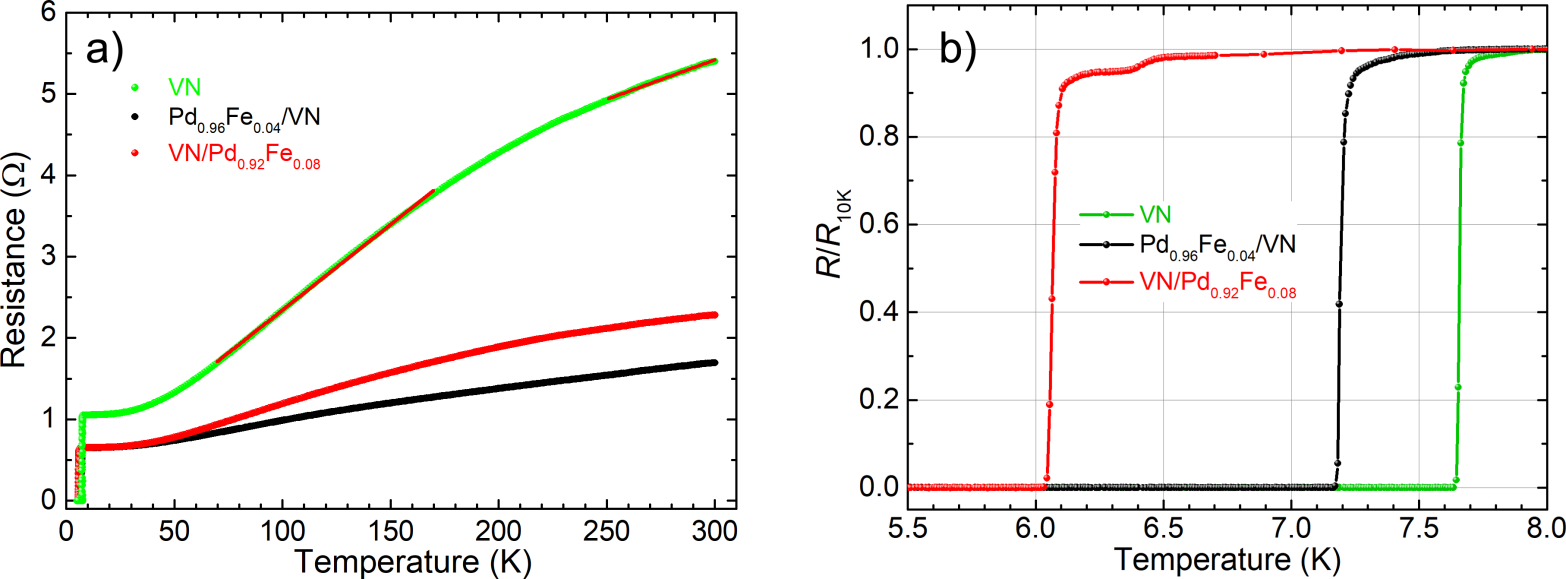
Temperature dependence of the electrical resistance of the VN film and the Pd_0.96_Fe_0.04_/VN and VN/Pd_0.92_Fe_0.08_ heterostructures: (a) full temperature range, (b) low-temperature region.

A physical property measurement system (QUANTUM DESIGN PPMS-9, USA) was used for studying the temperature dependence of the electrical resistance of the VN thin films and Pd_0.96_Fe_0.04_/VN and VN/Pd_0.92_Fe_0.08_ heterostructures in the temperature range of 4.2–300 K. A four-probe resistance measurement scheme was used. Figure 5 shows the measurement results as a function of the temperature for the epitaxial VN film and the heteroepitaxial Pd_0.96_Fe_0.04_/VN and VN/Pd_0.92_Fe_0.08_ samples. Table 1 contains the data on the residual resistance ratio RRR (i.e., the ratio of room temperature resistance, *R*_300K_, to the resistance at 10 K, *R*_10K_), the superconducting transition temperature (mid-transition criterion) and the width of the superconducting transition (10–90% criterion) for the VN thin film and the heterostructures with Pd_1−_*_x_*Fe*_x_*.

**Table 1:** Electrical and superconducting properties of the VN film and the Pd_0.96_Fe_0.04_/VN and VN/Pd_0.92_Fe_0.08_ heteroepitaxial structures on MgO(001).

structure	RRR	*T*_c_ (K)	Δ*T*_c_ (mK)

VN(30 nm)	5.2	7.7	25
Pd_0.96_Fe_0.04_(20 nm)/VN(30 nm)	3.5	7.2	37
VN(30 nm)/Pd_0.92_Fe_0.08_(12 nm)	2.6	6.1	50

The temperature dependence of the resistance of the VN thin film is of metallic type and exhibits two temperature intervals, one above 250 K and another one in the range of 80–180 K, of quasi-linear temperature dependence with different temperature coefficients of resistivity (TCR), i.e., 9.7 × 10^−3^ Ω/K and 2.1 × 10^−2^ Ω/K, respectively, marked by red straight lines over the green line in Figure 5a. It is similar to the *R*(*T*) behavior of VN/MgO(011) samples in [42], which was explained by a change in the electron/phonon scattering amplitude upon the structural phase transition from cubic to tetragonal at *T*_s_ = 250 K. Below 50 K the *R*(*T*) dependence saturates approaching the residual resistance originating, in general, from impurities and imperfections. Further cooling results in the phase transition to the superconducting state as it is shown in Figure 5b. The RRR value of 5.2 and the room-temperature resistivity of 42.5 μΩ·cm for the 30 nm thick VN film are among the best values obtained to date [42–45], indicating the high purity and structural quality of our VN film.

The superconducting transition temperature *T*_c_ of the VN film is 7.7 K (see Table 1), which is well above the temperature of liquid helium, LHe*T* = 4.2 K. Figure 5b shows a very sharp resistive transition at *T* = 7.7 K with a small width of 25 mK, which is quite remarkable compared to an elemental niobium film of the same (30 nm) thickness deposited in the same chamber and under vacuum conditions (Δ*T*_c_ [Nb(30 nm)]) = 10–23 mK).

Combining the VN film into a heterostructure with a palladium-rich Pd_1−_*_x_*Fe*_x_* alloy leads to a lowering of *T*_c_ because of the proximity effect [28]. This may shift the material operation temperature close to or even below the LHe*T*. With the iron content *x* in Pd_1−_*_x_*Fe*_x_* alloy below 0.08 its magnetic properties meet all the requirements for the F-layer in superconducting spintronic S/F/S-type structures, i.e., it is a weak ferromagnet with a low coercive field [41]. It is important that magnetic properties of epitaxial Pd_1−_*_x_*Fe*_x_* films are precisely controlled with the iron content *x* [41], and a perfect cube-on-cube epitaxy is realized with either the MgO(001) substrate or with the superconducting VN layers in any sequence. Figure 5b shows that 12 nm thick layer of Pd_0.92_Fe_0.08_ alloy adjacent to the 30 nm VN film lowers *T*_c_ from 7.7 K to 6.1 K, which is well above the LHe*T*. Moreover, Figure 5b demonstrates that the transition stays sharp: the maximum Δ*T*_c_ increases only to 50 mK, and there is no tail towards lower temperatures. Also, there is a room to optimize the superconducting parameters of the VN film towards an increase in *T*_c_ by about 1 K [43–44]. In our opinion, the results hint at a possible use of heteroepitaxial combinations of nitrides as superconductors and palladium-rich Pd_1−_*_x_*Fe*_x_* alloys as weak tunable ferromagnets to improve the operation characteristics of superconductor–ferromagnet–insulator heterojunctions for superconducting spintronics applications. For example, cubic superconducting MoN*_x_*, which is a Josephson junction technology material [4–5,46], exhibits a good epitaxial match with Pd_1−_*_x_*Fe*_x_* alloys, *a*_0_(MoN) = 416.3 pm.

## Conclusion

Fully epitaxial single-crystalline thin films of VN and heteroepitaxial structures of Pd_1−_*_x_*Fe*_x_*/VN and VN/Pd_1−_*_x_*Fe*_x_* (*x* = 0.04, 0.08, respectively) were grown on single-crystalline MgO(001) substrates using a combination of UHV molecular beam epitaxy and magnetron sputtering. The obtained 30 nm thick VN films exhibit a sharp superconducting transition with *T*_c_ = 7.7 K and Δ*T*_c_ = 25 mK. The heteroepitaxial Pd_0.96_Fe_0.04_/VN and VN/Pd_0.92_Fe_0.08_ structures reveal a superconductor–ferromagnet proximity suppression of the transition temperature to *T*_c_ = 6.1 K. This is, however, well above the liquid helium temperature of 4.2 K and, therefore, suitable for superconducting spintronics. The superconducting transition stays sharp with a somewhat larger width of Δ*T*_c_ = 50 mK. Moreover, there is no resistive tail towards lower temperatures. These results, in our opinion, indicate that fully epitaxial Pd_1−_*_x_*Fe*_x_*/VN and VN/Pd_1−_*_x_*Fe*_x_* thin film stacks can be considered as building blocks for superconducting spintronics elements.
